# Precision, Reliability, and Effect Size of Slope Variance in Latent Growth Curve Models: Implications for Statistical Power Analysis

**DOI:** 10.3389/fpsyg.2018.00294

**Published:** 2018-04-17

**Authors:** Andreas M. Brandmaier, Timo von Oertzen, Paolo Ghisletta, Ulman Lindenberger, Christopher Hertzog

**Affiliations:** ^1^Center for Lifespan Psychology, Max Planck Institute for Human Development, Berlin, Germany; ^2^Max Planck UCL Centre for Computational Psychiatry and Ageing Research, Berlin, Germany; ^3^Department of Psychology, University of Virginia, Charlottesville, VA, United States; ^4^Department für Psychologie, Universität der Bundeswehr München, Neubiberg, Germany; ^5^Faculty of Psychology and Educational Sciences, University of Geneva, Geneva, Switzerland; ^6^Faculty of Psychology (French), Swiss Distance Learning University, Brig, Switzerland; ^7^Swiss National Center of Competences in Research LIVES-Overcoming Vulnerability: Life Course Perspectives - University of Geneva, Geneva, Switzerland; ^8^Department of Political and Social Sciences, European University Institute, Fiesole, Italy; ^9^School of Psychology, Georgia Institute of Technology, Atlanta, GA, United States

**Keywords:** linear latent growth curve model, statistical power, effect size, effective error, structural equation modeling, reliability, longitudinal study design

## Abstract

Latent Growth Curve Models (LGCM) have become a standard technique to model change over time. Prediction and explanation of inter-individual differences in change are major goals in lifespan research. The major determinants of statistical power to detect individual differences in change are the magnitude of true inter-individual differences in linear change (LGCM slope variance), design precision, alpha level, and sample size. Here, we show that design precision can be expressed as the inverse of *effective error*. Effective error is determined by instrument reliability and the temporal arrangement of measurement occasions. However, it also depends on another central LGCM component, the variance of the latent intercept and its covariance with the latent slope. We derive a new reliability index for LGCM slope variance—effective curve reliability (ECR)—by scaling slope variance against effective error. ECR is interpretable as a standardized effect size index. We demonstrate how effective error, ECR, and statistical power for a likelihood ratio test of zero slope variance formally relate to each other and how they function as indices of statistical power. We also provide a computational approach to derive ECR for arbitrary intercept-slope covariance. With practical use cases, we argue for the complementary utility of the proposed indices of a study's sensitivity to detect slope variance when making *a priori* longitudinal design decisions or communicating study designs.

## Introduction

People differ in rates of change in many functional domains, both at behavioral and neural levels of analysis (e.g., Lindenberger, 2014). Describing, explaining, and modifying between-person differences in change are the central goals of lifespan research (Baltes and Nesselroade, 1979; Baltes et al., 2006; Ferrer and McArdle, 2010; McArdle and Nesselroade, 2014). Successful detection of inter-individual differences in change requires longitudinal (within-person) study designs with adequate statistical power.

Statistical power, the likelihood of rejecting a false null hypothesis, is a function of sample size, test size (that is, alpha level), population effect size, and the precision of measurement. In their considerations on optimal power in longitudinal designs, Rast and Hofer (2014) illustrated that Willett's (1989) reliability of the growth rate—as captured by Growth Rate Reliability (GRR)—plays an important role in determining statistical power in longitudinal designs using linear latent growth curve models. Latent growth curve models (LGCM) have become a commonly used analysis technique to capture change in longitudinal data (e.g., Meredith and Tisak, 1990; Ferrer and McArdle, 2003, 2010; Duncan et al., 2006). Here we formally show why GRR is a useful index of statistical power to detect slope variance in LGCM. However, we also reveal that GRR may provoke misleading interpretations because it remains constant over conditions that alter statistical power to detect individual differences in LGCM slopes using likelihood ratio tests. Specifically, the magnitude of stable individual differences (intercept variance) influences the power of 1-*df* likelihood ratio (LR) tests on slope variance. However, this is not reflected by GRR. Furthermore, the existence of intercept-slope covariance implies the existence of slope variance, yet GRR does not change as a function of intercept-slope covariance. These observations motivate and justify the development of a new, more comprehensive reliability measure that is coherent with the statistical power of LR tests.

Central factors affecting statistical power to detect individual differences in linear change include (a) the time elapsing from the beginning to the end of a study (henceforth referred to as total study duration); (b) the number of measurement occasions and their distribution over total study duration; (c) for each measured construct, the precision of the measurement instruments administered at each occasion; and (d) the number of participants, including any partial sampling designs, such as withholding assessments to estimate practice effects (Baltes et al., 1988), or other forms of planned missingness (McArdle, 1994; Wu et al., 2016). Simulations of statistical power to detect LGCM slope variances have already taken at least some of these factors into account when evaluating questions about statistical power (e.g., Hertzog et al., 2008; Wänström, 2009; Rast and Hofer, 2014; Ke and Wang, 2015).

In this article, we present a formal derivation of measures of precision, reliability, and statistical power that conform to LR tests of slope variance. All three types of measures can be regarded as gauging a design's sensitivity to detect individual differences in linear slopes (henceforth simply referred to as *change sensitivity*). We derive a new measure of reliability, which we term *effective curve reliability* (ECR). ECR can be regarded as a standardized effect size measure that is coherent with power to detect individual differences in linear change using LR tests. We formally outline the conditions under which GRR and ECR are identical, as well as the conditions under which they diverge. We also discuss potential applications of such measures in the service of *a priori* longitudinal design decisions.

## Effective error and effect size for slope variance

### Linear latent growth curve models

A linear LGCM for balanced designs assumes linear growth in a variable *x*_*ij*_ over time points *t*_*j*_ with *j* = 1*, …, M* on *i* = 1*, …, N* persons with inter-individual differences in the intercept and the linear slope, which are represented by latent variable means and (co)variances. In a linear LGCM, the mean vector, μ, and the covariance matrix, Σ, of the observed variables are a function of factor loadings, Λ, latent variables' intercepts, ν, a latent variance-covariance matrix, Ψ, and a residual variance-covariance matrix, Θ (e.g., Bollen, 1989):

(1) $$\begin{array}{l} \Sigma =\Lambda \Psi \Lambda^{\prime }+\Theta \\ \;\mu =\Lambda \nu \end{array}$$

Under the assumption of homoscedastic and uncorrelated residual errors, we define

(2) $$\begin{array}{l} \Lambda =\left[\begin{array}{cc} 1 & t_{1}\\ 1 & t_{2}\\ 1 & \vdots \\ 1 & t_{M} \end{array}\right]\\ \Psi =\left[\begin{array}{cc} \sigma_{I}^{2} & \sigma_{IS}\\ \sigma_{IS} & \sigma_{S}^{2} \end{array}\right]\\ \nu =\left[\begin{array}{c} \mu_{I}\\ \mu_{S} \end{array}\right]\\ \Theta =\left[\begin{array}{ccc} \sigma_{\epsilon }^{2} & 0 & 0\\ 0 & \ddots & 0\\ 0 & 0 & \sigma_{\epsilon }^{2} \end{array}\right] \end{array}$$

with the number of measurement occasions *M* at times *t*_1_ to *t*_*M*_, the homogeneous residual error $\sigma_{\epsilon }^{2}$, the mean μ_*I*_, and variance $\sigma_{I}^{2}$ of the latent intercept, and the mean μ_*S*_, and the variance of the latent slope $\sigma_{S}^{2}$, and the latent intercept-slope covariance σ_*IS*_. One of the *t*_*j*_*, j* ∈ {1*, …, M}* can be conveniently fixed to zero to identify the slope and intercept. Often *t*_1_ is fixed to 0 to define the intercept at the first occasion of measurement (*j* = 1), but other choices are possible.

In principle, SEM, multi-level model (e.g., Goldstein, 1986), and random-effects model (e.g., Laird and Ware, 1982) representations of the LGCM are empirically and analytically identical (e.g., Rovine and Molenaar, 2000; Curran, 2003). For the sake of clarity, we restrict our perspective to a treatment from a SEM approach but our results and conclusions equally apply to algebraically equivalent growth curve modeling approaches.

### Specific and generalized variance tests

LGCM captures individual differences in rates of change in the between-person variance in linear slopes (e.g., Singer and Willett, 2003). In the univariate case, a formal statistical test of the null hypothesis of zero slope variance can be obtained by two types of LR χ^2^-tests. Each of the tests is based on the difference in −2LL (log-likelihood) goodness of fit of two nested models (meaning that the parameters of the simpler model represent a subset of the parameters of the larger model):

- A restricted model specifying that Ψ has a free intercept variance ($\sigma_{I}^{2}$) but fixing both the slope variance ($\sigma_{S}^{2}\;=\;0$) and the intercept-slope covariance (σ_*IS*_ = 0), and a second model freely estimating all three parameters in Ψ. This 2-*df* LR test relies on information in both the slope variance and intercept-slope covariance to test the null hypothesis of zero population slope variance.
- An alternative 1-*df* LR test ignores the information in the intercept-slope covariance (by assuming it to be zero in the less restricted model) and tests the loss of information by fixing only the variance (that is, $\sigma_{S}^{2}\;=\;0$) in the more restricted model.

In practice, a third test is more often employed, a 1-*df* Wald test (Bollen, 1989), generated from an estimated LGCM solution: the estimated slope variance divided by its estimated standard error of estimate. There are principled reasons in actual practice to prefer the 2-*df* LR test over the 1-*df* LR test or the Wald test (see section Discussion), but the 1-*df* LR test is particularly useful for change-sensitivity analysis in longitudinal research design when intercept-slope covariance is not considered substantial.

### Prelude on change sensitivity and statistical power

How can one best examine the sensitivity of linear change measurements? von Oertzen (2010) and von Oertzen and Brandmaier (2013) developed the concept of *effective error*, which captures the precision with which properties of the latent slope in a LGCM can be measured. In a single measurement, measurement error of an instrument quantifies the amount of unsystematic variance (and its inverse quantifies the precision of measurement). In a longitudinal design, effective error variance quantifies the magnitude of unsystematic variance in the outcome of interest over repeated measures (and its inverse is proportional to the precision of the repeated measures design to measure a given outcome). Effective error is thus a measure of sensitivity to detect a hypothesized effect. It can be computed as a weighted composite of all variance sources in a LGCM that potentially distort the measurement of the effect of interest. As we will show below, major components of effective error for LGCM linear slope variance are the temporal arrangement of measurement occasions and instrument reliability. Effective error is also scaled by intercept variance expressing the stable between-person differences. As such, effective error is primarily an index of change sensitivity that requires no assumption about the true absolute (or, unstandardized) effect size, sample size, or test size (significance level of the statistical test). Conceptually, effective error can be construed as the error a researcher would have experienced if one were able to measure the latent construct of interest (here, variance in linear change) with a single measurement. Despite the fact that this one-shot measurement of change can never be attained in reality, it can be shown that the corresponding minimal study design has equivalent statistical power to the original study design (von Oertzen, 2010).

**Figure 1:**
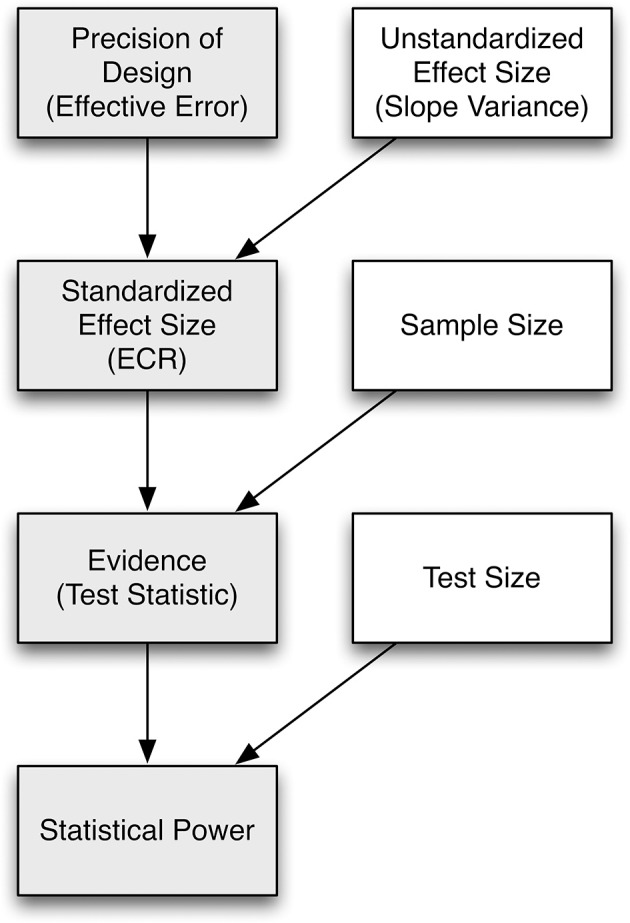
The relation of effective error (as the inverse of precision of a study design), reliability, standardized effect size, evidence, and statistical power is shown as a dependency graph. Gray boxes indicate measures, and white boxes indicate design factors or population values. Each measure conveys change sensitivity, with the number of assumptions to be made about design factors and population values increasing from top to bottom.

How is the concept of effective error relevant for understanding statistical power? Recall that classic treatments of power consider two main parameters that govern the power of a study to reject the null hypothesis about a parameter value when it is false (e.g., Cohen, 1988): effect size (as it relates to the non-centrality parameter of the sampling distribution), and dispersion of the sampling distribution, with two main constituents: sample size and “error variance.” Consider the dependency graph in Figure 1, which we use as a scheme to develop a hierarchical conceptualization of change sensitivity. We start with effective error and arrive at statistical power. From top to bottom, the measures require an increasing number of assumptions to be made about design factors or population values (represented as white boxes): measurement precision, true effect size, and test size. At the top of the hierarchy, effective error is a measure of precision of a repeated-measures design, construed broadly to imply all sources of variance that contribute to the dispersion in the sampling distribution of the test statistic. If an unstandardized population effect size (i.e., the absolute value of the population slope variance parameter) is known or assumed, one can derive a standardized effect size that rescales the parameter against effective error to generate a metric-free standardized effect size. Put differently, standardized effect size can be conceptualized as the reliability of the population effect, for instance, slope variance, in relation to the precision with which it can be measured (e.g., Willett, 1989; Kelley and Preacher, 2012). When scaling the standardized effect size with sample size, we obtain *evidence* (the magnitude of evidence against the null hypothesis) as a measure of change sensitivity. Once one is willing to determine criteria of the statistical test (i.e., the Type I error rate), statistical power itself is possibly the ultimate index of change sensitivity as it determines the probability of correctly rejecting the null hypothesis when it is false. We stress that no single measure of change sensitivity is generally superior to any other; instead, they complement each other and convey different perspectives to change sensitivity, and each is based on different population values or design factors (see Figure 1).

### Effective error of the specific variance test

The analytical derivations of effective error, standardized effect size, and statistical power in this paper are based on the assumption of no intercept-slope covariance, which renders them coherent with the assumptions of the specific 1-*df* LR test of the null hypothesis of zero slope variance. When the true intercept-slope covariance parameter is different from zero, the 2-*df* test should be used because (a) it has greater power to reject the null hypothesis (Pinheiro and Bates, 2000; Hertzog et al., 2008), and (b) the 1-*df* specific variance test is misspecified, as it represents a LR test of two restricted models both incorrectly specifying zero intercept-slope covariance. Currently, there is no known closed-form solution for ECR in the general case. However, in section Generalized ECR, we will provide an algorithm to compute the ECR numerically for cases in which the intercept-slope covariance is non-zero. This yields a standardized effect size metric coherent with the statistical power of the 2-*df* generalized variance test. We proceed by presenting the analytical solutions of precision, reliability, and power for the 1-*df* test. This test is not only practically relevant but enables us to set out a comprehensive perspective on the roles of GRR and ECR in determining statistical power to detect individual differences in linear change.

**Figure 2:**
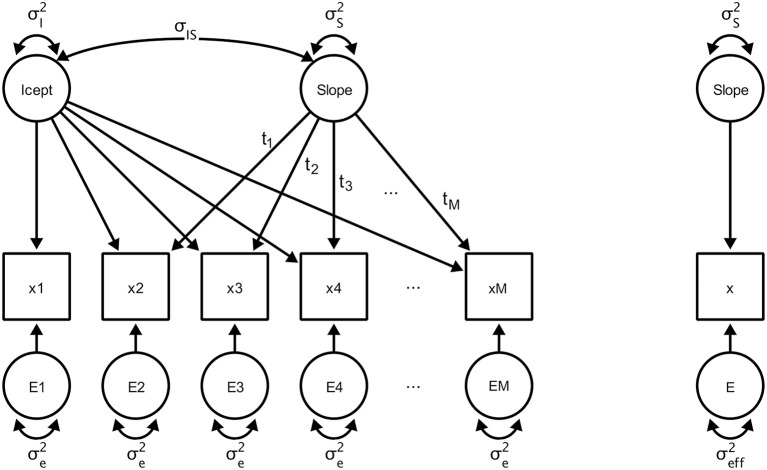
**Left**: A LGCM with a latent intercept “Icept” with variance $\sigma_{I}^{2}$ and a latent slope “Slope” with variance $\sigma_{S}^{2}$, and intercept-slope covariance σ_IS_. A single variable is observed for a total of *M* occasions of measurement (labeled x1 to xM) and an error term with variance $\sigma_{e}^{2}$. **Right**: A hypothetical minimal and power-equivalent model representing a design in which the slope is measured directly with a single source of effective error.

We now review a formal solution that allows one to derive the proposed measures of change sensitivity, starting with the variance of the effective error, for tests of linear slope variance in a longitudinal study. We use power-equivalent^1^ transformations, defined as transformations that structurally change the model but preserve statistical power for a given hypothesis (see MacCallum et al., 2010; von Oertzen, 2010), to transform the original complex design into a power-equivalent model with minimal parameterization (see Figure 2). The resulting condensed model specifies direct observation of the slope variance (which is, of course, not possible in reality) and its corresponding residual, effective error, which captures the aggregate effects of all variables on precision of measurement.

Effective error serves as a basis for comparison of change sensitivity across multiple studies that differ in specification (von Oertzen and Brandmaier, 2013). The major practical value of an expression for effective error is that models with equal effective error have equal power to reject the null hypothesis of non-zero slope variance, even if they are structurally different (under the assumption that all parameters but the slope variance are assumed fixed; see von Oertzen, 2010).

Here, we argue in favor of measures of change sensitivity that are coherent with power for the specific variance test, that is, a test that assumes no intercept-slope covariance (see *null model* in Section Specific and generalized variance test). Extending previous work (von Oertzen and Brandmaier, 2013), we derive a general-case, analytical solution of effective error for known and fixed non-zero intercept-slope covariance (see section 1 in Supplementary Material). In the simplifying case of no intercept-slope covariance, we obtain:

(3) $$\begin{array}{r} \sigma_{eff}^{2}=\frac{\sigma_{\epsilon }^{2}}{\overset{M}{\underset{j=1}{\sum }}t_{j}^{2}-\eta {\left(\overset{M}{\underset{j=1}{\sum }}t_{j}\right)}^{2}} \end{array}$$

with

(4) $$\begin{array}{c} \eta =\;\frac{1}{M+\frac{\sigma_{\epsilon }^{2}}{\sigma_{I}^{2}}}=\frac{\sigma_{I}^{2}}{M(\sigma_{I}^{2}+\sigma_{\epsilon }^{2}/M)}\;=\;\frac{ICC_{2}}{M} \end{array}$$

and $\sigma_{\epsilon }^{2}$ being the LGCM residual variance, $\sigma_{I}^{2}$ being the intercept variance, *t*_*j*_ being the time point of measurement occasion *j* (varying from 1 to *M*) and *ICC*_2_ being the intra-class correlation coefficient for M repeated measures.

As can be seen from Equations (3) and (4), the effective error depends directly on ICC_2_, and, thus, also on intercept variance $\sigma_{I}^{2}$. Effective error increases with increasing ICC_2_ such that change sensitivity decreases when intercept variance is large in comparison to residual variance, or in other words, change sensitivity increases with the amount of shrinkage captured as ICC_2_. In summary, effective error increases with residual error variance $\sigma_{\epsilon }^{2}$, increases with intercept variance $\sigma_{I}^{2}$, decreases with the number of measurement occasions *M*, and quadratically decreases with the measurement time points *t*_*j*_, corresponding to an asymptotic decrease proportional to the variance of the distribution of measurement occasions.

## Reliability of random slopes in LGCM

Reliability is typically conceived as a measure of consistency, precision, or repeatability of measurement in a population (Kline, 1998). In practice, a reliable measurement instrument reproduces the same or similar scores when measuring the same construct from one assessment to another. This was the principal justification used by Willett (1989) in deriving GRR, which he treated as an extension of the reliability of a simple difference score to cover the reliability of slope variance across larger numbers of measurement occasions. We take a similar approach here, but directly link the concept of reliability of the slope variance to effective error, grounding that concept in the basis for statistical power of the LR test of slope variances. In a hypothetical model, in which slope variance could be directly measured, total observed slope variance would be due to true score variation, that is, individual differences in linear slope and measurement error variance.

We depart from Willett (1989) in the error term used to scale reliability of measurement for LGCM slopes, using the effective error $\sigma_{eff}^{2}$ instead, to rescale the true effect of inter-individual differences $\sigma_{S}^{2}$ in order to yield an alternative reliability index for slopes, *effective curve reliability* (ECR). ECR scales slope variance as a proportion of the sum of slope variance and slope measurement error:

(5) $$\begin{array}{r} ECR=\frac{\sigma_{S}^{2}}{\sigma_{S}^{2}+\sigma_{eff}^{2}} \end{array}$$

ECR is thus an index that ranges between zero and one, and becomes larger as population slope variance increases, or as the precision of the longitudinal study design reduces effective error. Conceptualized as reliability, it can also be regarded as a standardized effect size statistic (Kelley and Preacher, 2012).

When intercept-slope covariance is negligible, ECR is obviously similar to the aforementioned GRR. In fact, GRR is related to ECR and equal to it in a few limiting cases. We illustrate the relations of ECR and GRR using the equations above that assume that σ_*IS*_ = 0. One noteworthy limiting case is when ICC_2_ = 1, where individual differences account for all of the reliable sources of variance in the dependent variable over time; that is, each measurement within a person strongly depends on the others and the information gained about intercept differences by any given measurement within one person is largely redundant:

(6) $$\begin{array}{r} {\tilde{\sigma }}_{eff}^{2}=\frac{\sigma_{\epsilon }^{2}}{SST} \end{array}$$

with *SST* being the squared deviations of t_j_ about their mean. Formally:

(7) $$\begin{array}{r} SST\;=\;\overset{M}{\underset{j\;=\;1}{\sum }}{\left(t_{j}-\bar{t}\right)}^{2}\text{ and }\bar{t}\;=\;\overset{M}{\underset{j\;=\;1}{\sum }}\frac{t_{j}}{M} \end{array}$$

Inserting this special-case effective error, ${\tilde{\sigma }}_{eff}^{2}$, into the definition of ECR, we obtain GRR:

(8) $$\begin{array}{r} GRR\;=\;\frac{\sigma_{S}^{2}}{\sigma_{S}^{2}+\sigma_{\epsilon }^{2}/SST} \end{array}$$

A second limiting case is when ICC_2_ = 0 renders η = 0 in Equation (3), and produces a similar simplification on effective error. It renders ECR = GRR because there is no intercept variance. Otherwise, ECR and GRR diverge by the degree to which ICC_2_ is smaller than one, that is, to the extent that intercept variance is relatively small or residual error variance large. As a final special case, GRR = ECR when the sum across time points is zero, ∑*t*_*i*_ = 0, which is the case in models in which the intercept is centered with respect to *t*:

(9) $$\begin{array}{r} \sigma_{eff}^{2}=\frac{\sigma_{\epsilon }^{2}}{\overset{M}{\underset{j\;=\;1}{\sum }}t_{j}^{2}-\eta \cdot {\left(\overset{M}{\underset{j\;=\;1}{\sum }}t_{j}\right)}^{2}}=\frac{\sigma_{\epsilon }^{2}}{\overset{M}{\underset{j\;=\;1}{\sum }}t_{j}^{2}}=\frac{\sigma_{\epsilon }^{2}}{SST}={\tilde{\sigma }}_{eff}^{2} \end{array}$$

Otherwise, ECR will differ from GRR. As we show next, ECR's direct connection to intercept variance ensures that it, and not GRR, is coherent with power of the 1-*df* LR test for slope variance.

### Monte Carlo simulation of ECR, GRR, and power of the 1-*df* slope variance test

**Figure 3:**
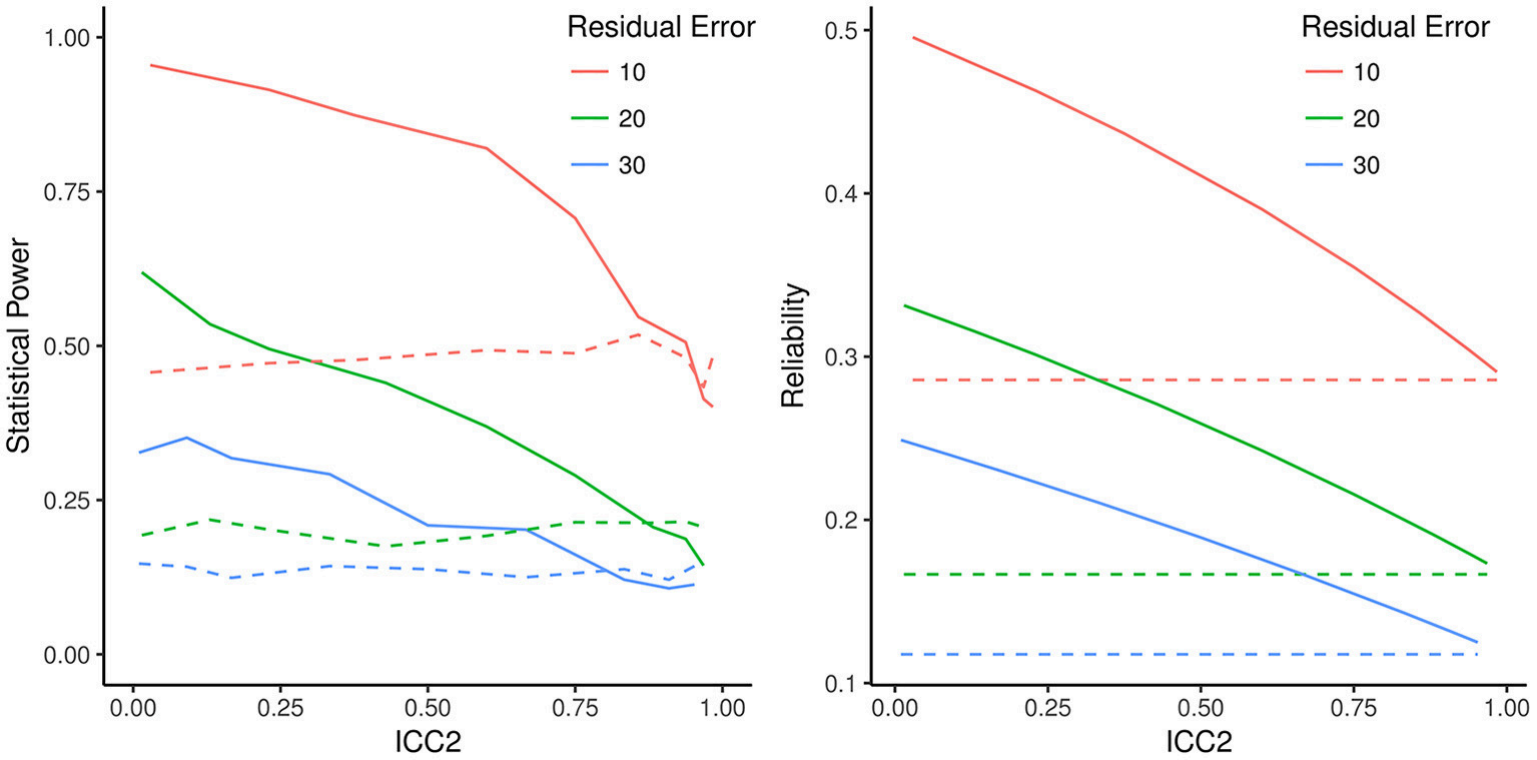
Statistical power for specific variance test and Wald test, and effective curve reliability (ECR) and Growth Rate Reliability (GRR), as an effect of varying intercept variance scaled as the intra-class correlation coefficient (ICC_2_). **Left**: Lines are Monte Carlo estimates of statistical power of the specific variance test (solid) and Wald test (dashed) with each line representing a model with different residual error variance. **Right**: Corresponding ECR (solid lines) and GRR (dashed lines). GRR is constant for all intercept variance values (scaled as ICC_2_) whereas ECR traces the shape of change in statistical power as seen in the left panel. GRR and ECR converge to the same value as ICC_2_ approaches 1.

To illustrate that the statistical power of the 1-*df* LR test is dependent on intercept variance, as expected based on the definition of effective error, we ran selected Monte Carlo simulations manipulating intercept variance. We hypothesized that the effect of intercept variance on the power of the LR test is captured by ECR but not by GRR. We also simulated the Wald test as defined in section Specific and Generalized Variance Tests. The baseline model for the simulations had three measurement occasions with equally spaced intervals and a sample size of 100 (cf. Figure 3). For illustration, slope variance was set to 2, residual variance was varied between 10, 20, and 30 (as indicated by the different lines) and intercept variance was varied between 0.1 and 200 to cover a wide range of possible ICC_2_ values. The intercept was defined at the first occasion of measurement by fixing the basis vector loading *t*_1_ to zero. The critical purpose of the simulation is to show the effect of ICC_2_ on power of the 1-*df* LR test and the Wald test.

Figure 3 depicts the simulation results based on 1,000 Monte Carlo replications of each condition. The left-hand panel plots power as a function of varying intercept variance scaled as ICC_2_. Clearly power of the specific variance LR test (solid lines) is strongly affected by the proportion of intercept variance, as predicted. However, this is not the case for the Wald test (dashed lines). The right-hand panel plots ECR and GRR as a function of the same ICC_2_. GRR, plotted in dashed lines, is unaffected by ICC_2_. In contrast, ECR (solid lines of right-hand panel) tracks the effects of ICC_2_ on estimated power. ECR does not ignore intercept variance and thus conforms to the effective error and power of the 1-*df* LR test, whereas GRR is in line with the Wald test, which ignores intercept variance. The simulation shown in Figure 3 is grounded in a particular set of LGCM parameter values, but reflects a general principle of coherence of ECR with power under the given assumptions.

**Figure 4:**
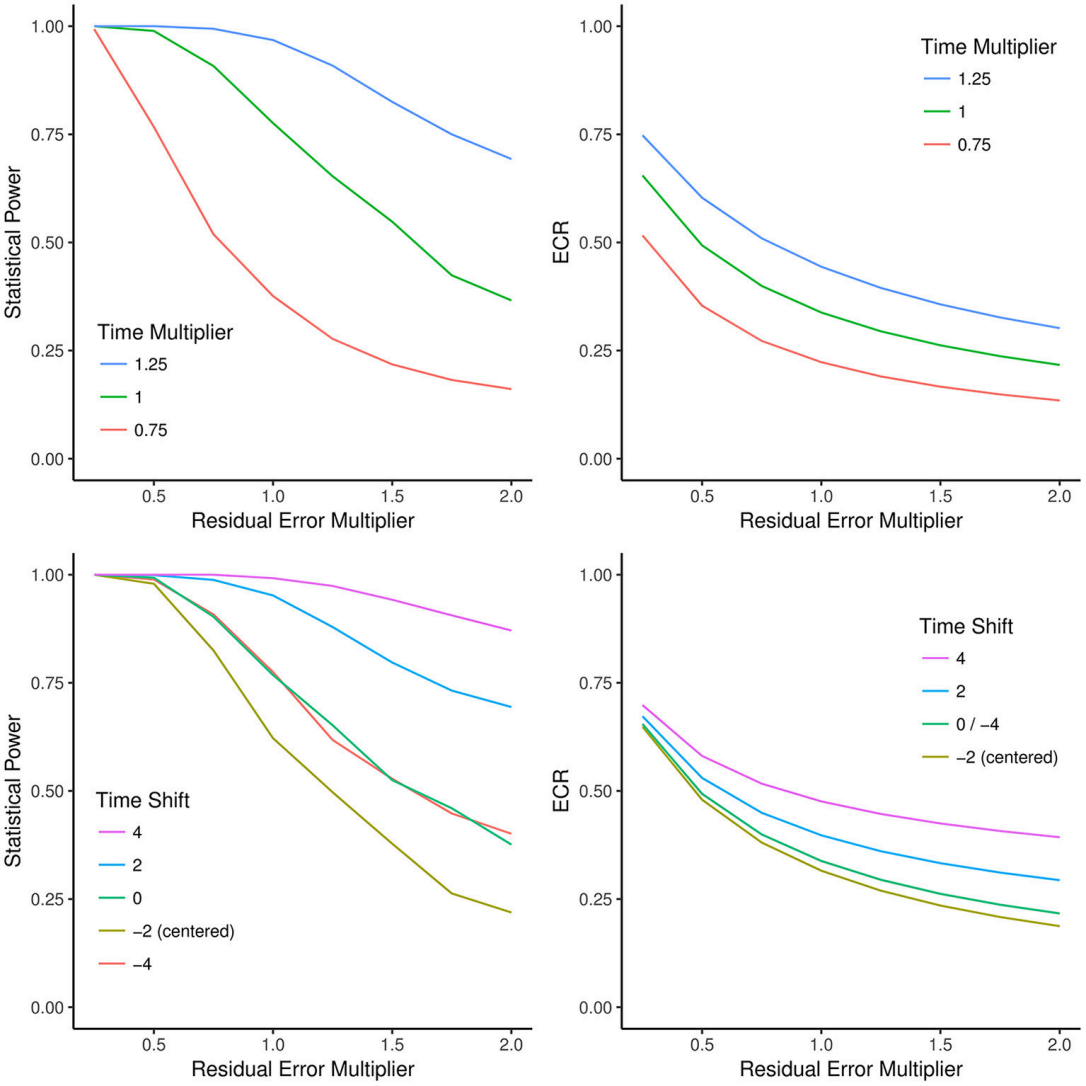
The effects of shifting, prolonging, or compressing the study duration on statistical power and effective curve reliability (ECR). **Top left**: Monte Carlo simulation of the effect of decreasing or increasing residual error variance (x-axis) or shortening/prolonging total study duration (blue, green, and red lines) on statistical power (y-axis). **Top right**: The effect of decreasing or increasing residual error variance (x-axis) or shortening/prolonging total study duration (blue, green, and red lines) on ECR (y-axis). ECR traces the same shapes of decreases in statistical power as observed in the **top left** panel. **Bottom left**: Monte Carlo simulation of the effect of shifting the measurement time points by factor (−4, −2, 0, 2, 4; colored lines) and changing residual error variance (x-axis) on statistical power (y-axis). Note that anchoring the intercept at the first or last measurement has identical power. Lowest power is achieved with a centered design. Bottom right: The effect of shifting the measurement time points by factors (−4, −2, 0, 2, 4; colored lines) and changing residual error variance (x-axis) on ECR (y-axis). Note that the green line represents both the original design (shift: 0) and a design with intercept variance anchored at the last measurement (shift: −4), both of which empirically have identical power whereas the centered design has lowest ECR and power. As shown in Equation (11), for all variations in time sampling, GRR yields the values corresponding to the ECR of the time-centered design (yellow line; *t* = −2), and thus differential power because time shifts are not reflected in GRR.

In Figure 4, we extended this simulation to show how statistical power and ECR change as a function of either shifting measurement time points in time (including centering the design) and prolonging or shortening total study duration by a multiplicative factor. The baseline model is a linear change model with three measurement occasions, baseline, 2 years after, and 4 years after beginning of the study. Intercept, slope, and residual variance were modeled after the values reported by Rast and Hofer (2014) for the *Memory-in-Reality* measure from the OCTO-Twin study (Johansson et al., 1999, 2004). Figure 4 shows that both ECR and statistical power increase when total study duration is increased as measurement occasions are added. Importantly (but not shown in the figure), GRR does not change as a function of time shift (because *SST* is constant under time shift).

In summary, both ECR and GRR connect closely to statistical power to detect non-zero slope variance in LGCMs because they are standardized effect size measures for the slope variance, which makes them independent of the original metric in the dependent variable. Under the assumption of no intercept-slope correlation and if one wanted to use a Wald test, GRR is the appropriate effect size statistic. However, ECR is superior to GRR when computing effect sizes for the 1-*df* specific variance test because it traces statistical power more accurately.

If one assumes for the sake of argument that the selection of studies by Rast and Hofer (2014) is representative for a particular future longitudinal study of individual differences in psychological aging, one can compare expected differences between ECR and GRR in the range of plausible parameter values from their reported data sets. We used the reported data from Rast and Hofer (2014) and observed ICC_2_ between 0.62 and 0.98 (median: 0.89), which may—depending on the density of measurements—lead to substantial differences in the predictions about change sensitivity as captured by ECR vs. GRR. That is, within the reported range of the studies used in their meta-analysis, *post-hoc* GRR estimates range from 0.02 to 0.72 (median = 0.36) and ECR estimates range from 0.03 to 0.76 (median = 0.40).

Below, we illustrate that this magnitude of difference can have a serious impact on design decisions. Before doing so, we corroborate our perspective on the usefulness of ECR as a proxy for statistical power by formally showing how ECR relates to statistical power in the specific variance test.

## Formalizing the connection of ECR to statistical power of the 1-*df* slope variance test

In the typical fourfold table capturing hybrid Neyman–Pearson inference, the probability of Type I errors is denoted α and that of Type II errors is denoted as β. The power of a LR test (1 – β) to correctly reject a (false) null hypothesis is defined as the probability of obtaining a value of the test statistic that is larger than a critical value derived from the sampling distribution of that test statistic under the null hypothesis:

(10) $$\begin{array}{r} 1-\beta =\int_{\chi_{\nu ,0}^{2}\left(\alpha \right)}^{\infty }{\chi^{2}}_{\nu ,\lambda }\left(x\right)dx \end{array}$$

with $\chi_{\nu ,\lambda }^{2}$ being a non-central χ^2^-distribution with ν degrees of freedom and non-centrality parameter λ, and $\chi_{\nu ,0}^{2}\left(\alpha \right)$ denoting the *critical value* for test size α. Note that the start of the integral is the critical value for the null distribution, which is usually a central χ^2^-distribution with ν degrees of freedom. However, if the variance is at a boundary (i.e., in our situation, if the slope variance is restricted to be positive), this distribution may be different, e.g., a mixture of χ^2^-distributions (see Self and Liang, 1987; Stoel et al., 2006). Ignoring the mixture distribution may lead to lower power than one could achieve when attending to it (but see Kolenikov and Bollen, 2012). However this pertains equally across all possible designs and, thus, can be ignored at the level of effective error and reliability.

For a test of a single parameter, in particular, a restriction on the slope variance, we set ν = 1. The left integral bound χ^2^(ν, 0) is then determined by the critical value α as chosen by the investigator. The area to be integrated over depends on the non-centrality parameter λ, which again depends on the true slope variance, and effective error here. Using the approximation for λ by Satorra and Saris (1985), we obtain its value from the likelihood ratio of the minimal model with the slope variance restricted to zero over the minimal model without any restriction^2^. The model-predicted covariance of the unrestricted model is the sum of true slope variance and effective error, $\Sigma_{min}\;=\;\left[\sigma_{S}^{2}\;+\;\sigma_{eff}^{2}\right]$. The restricted minimal model assumes zero slope variance, which yields $\Sigma_{res}\;=\;\left[\sigma_{eff}^{2}\right]$. Under the assumption of an unrestricted mean structure, the log-likelihood ratio (see section 2 in Supplementary Material) as the estimate of the non-centrality parameter is

(11) $$\begin{array}{r} \lambda =N\left[\frac{1}{1-ECR}-\ln\;\left(\frac{1}{1-ECR}\right)-1\right] \end{array}$$

Omitting third-order terms, an approximation of this Equation is given by $\lambda \approx N\left(\frac{ECR^{2}}{1-ECR}\right)$ which reveals that the relation of ECR and λ is dominated by a quadratic term of reliability and a linear term of sample size.

To summarize, by calculating the effective error $\sigma_{eff}^{2}$ from the structural design parameters of a given LGCM (residual variance, time points of occasions of measurement, and intercept variance) and assuming a true slope variance $\sigma_{S}^{2}$, a sample size *N*, and a test size α, we can derive λ, the non-centrality parameter of the χ^2^-distribution under the hypothesis H_1_. By numerically integrating this χ^2^-distribution between a left bound depending on α, and infinity, we obtain the statistical power for the specific variance test under the assumption of all parameters but slope variance known and fixed. Note that under this assumption, the analytical power value is typically overestimated as freely estimating the remaining parameters will cost power. Still, it can be obtained that ECR and sample size are the primary determinants of statistical power of the LR test. The minimal power-equivalent metrics, effective error and ECR, are particularly useful as they constitute projections of the multidimensional design parameter space of a LGCM to a univariate index. They can both serve as comprehensive indices of change sensitivity in a longitudinal design exercise. ECR, in particular, is suitable as a general-purpose measure of slope effect size that is comparable across different studies because it does not depend on the units of measurement of the dependent variable.

### Generalized ECR

When intercept-slope covariance is non-zero, the 1-*df* specific variance test is mis-specified because it assumes a zero covariance $\sigma_{IS}^{2}\;=\;0$, and the analytical solution of ECR as presented above, which does not capture this misspecification, is no longer fully coherent with its power. Currently, there is no analytical solution to compute ECR from a set of LGCM parameters for arbitrary intercept-slope covariance, which is then coherent with the 2-*df* generalized variance test. However, we provide an algorithm that can be used to compute ECR in the general case (see section 3 in Supplementary Material). This 2-*df* ECR can be interpreted as a standardized effect size of the total latent information about individual differences in linear slope. Under the assumption that $\sigma_{IS}^{2}\;=\;0$, the analytical and computational solutions are identical.

**Figure 5:**
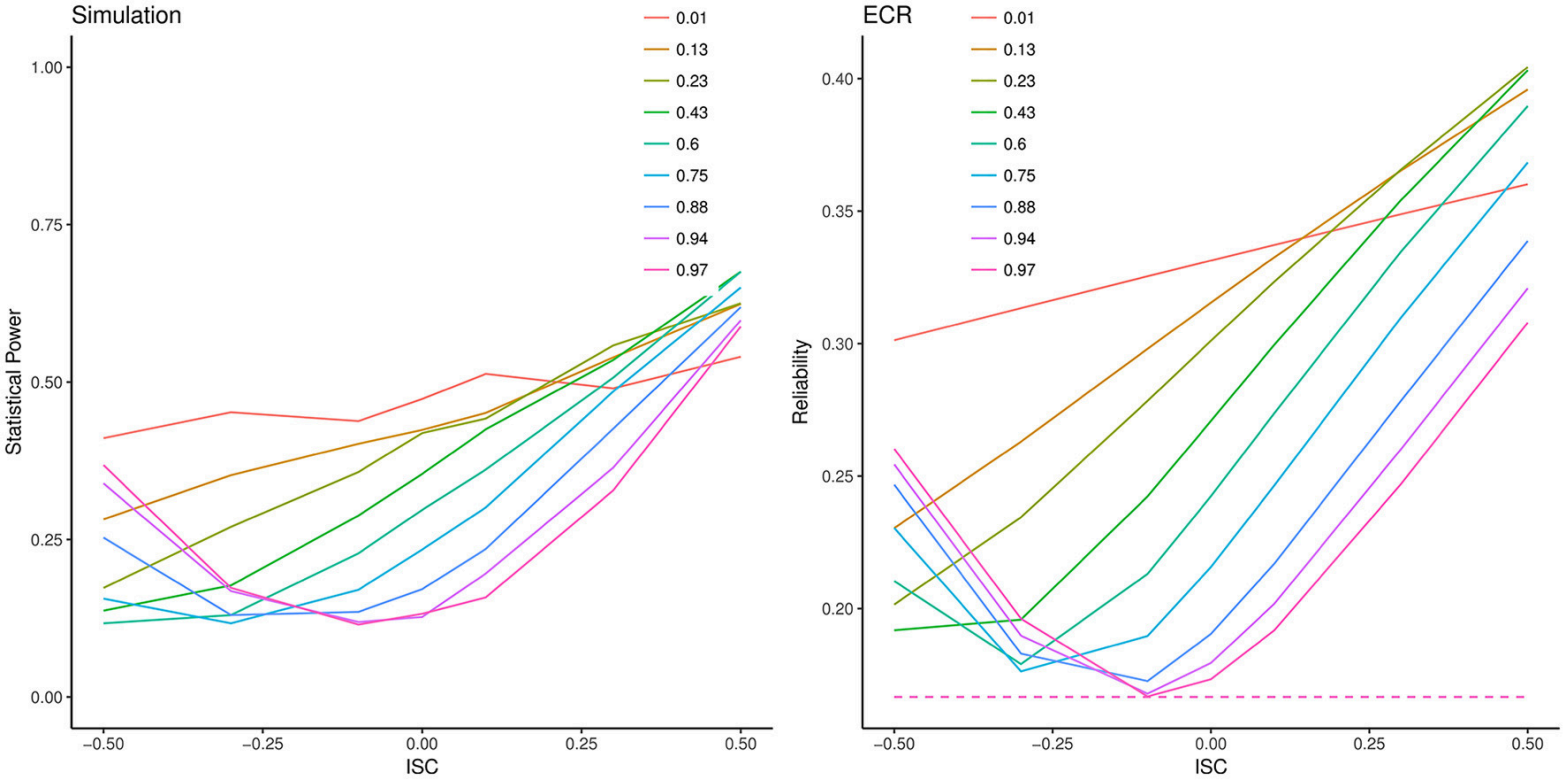
Statistical power and effective curve reliability (ECR) values for the 2-df generalized variance test as a function of intercept-slope correlation (ISC). **Left**: Lines are Monte Carlo estimates of statistical power of the generalized variance test, with each line representing a model with different intercept variance scaled as the intra-class correlation coefficient (ICC_2_). **Right**: Corresponding ECR (solid lines). GRR is constant for all conditions shown here.

To illustrate the coherence of this generalized index of ECR with power to detect slope variance, we ran an extension for one condition of our previous simulation considering the effect of intercept-slope covariance. Figure 5 shows a slice of the simulation where residual error variance was set to 20, As before, we varied ICC_2_ and also varied intercept-slope correlation, σ_*IS*_, between −0.5 and +0.5. The left-hand panel of Figure 5 shows the power of this test as a function of ICC_2_ (as in Figure 3), but also as a function of σ_*IS*_. The right-hand panel also displays the computed ECR as a function of ICC_2_ and σ*_IS_*. Clearly power of the 2-*df* generalized variance test is strongly affected by the proportion of intercept variance and the intercept-slope correlation. Most noteworthy, comparison of the two panels shows that ECR completely tracks these variations in power. In contrast, GRR (dashed line) is identical across all conditions shown, despite the fact that the observed power ranges between 10% and 70%. Clearly ECR, not GRR, is the appropriate standardized effect size index for the 2-*df* test.

### Considerations when gauging change sensitivity

We now consider the practical implementation of the concepts introduced above for the purpose of *a priori* power analysis when designing longitudinal studies. To illustrate our analytical derivations, we continue to assume no intercept-slope correlation, σ_*IS*_ = 0, but the same considerations can be made using the computational approach allowing for non-zero intercept-slope correlations. Let us assume that a developmental researcher is engaged in design decisions when planning a longitudinal study. In practice, one cannot necessarily use existing studies to fully determine the configuration of LGCM parameters that are needed to specify the statistical power of a given design precisely. One reason for this claim is that phenomena do not always generalize across different constructs and measures, but another is that published papers often do not report all the relevant parameter estimates that are needed to compute ECR. Instead, partial or indirect evidence regarding parameters is often reported, such as an estimate of slope variance but none of the remaining parameters. How should one proceed on the basis of whatever minimal information is available in advance about the magnitude of individual differences in change so as to inform the study design process and its sensitivity to detecting said change? Although there are no incontrovertible general answers to this question, there are a number of practical guidelines and solutions available depending on what is considered to be known about the phenomena of interest. In the following, we present several cases showing how one can make use of minimal information about measures and their change to generate feasible study designs.

The parameters that determine ECR and hence power to detect LGCM slope variances can be divided into two categories: those that describe the process under investigation and those that describe the study design. The former parameters determine the structural model (Ψ; see Equation 2) of the LGCM, that is, the slope variance, intercept variance, and intercept-slope covariance. The latter parameters comprise the measurement model of the LGCM (Λ and Θ; see Equation 2), and consist of measurement precision, number and temporal distribution of measurement occasions, and total study duration. For the most part, the parameters of the measurement model can be directly influenced by design decisions. Hence variations in those parameter values can and should be selected in ways that are appropriate to design pragmatics and inevitable external constraints, such as budget and time (see Brandmaier et al., 2015), while optimizing power to detect the effect of interest. Making informed choices about population values is typically more difficult but equally important, as the choice of these values will also influence the outcome of a power analysis for a given design problem. In particular, deriving a plausible estimate of the population slope variance for a given measure or set of measures is crucial because it fundamentally determines the ECR and hence statistical power. If estimates of unstandardized slope variance or values of ECR from previous studies are available, they likely provide the basis for a good first guess. We would therefore like to emphasize the pressing need to report such indices of change sensitivity. If necessary, a conservative best-guess must be made from whatever other information is available. According to Willett (1989), the most logical strategy is probably to adopt a specific target reliability, such as GRR or ECR, when planning a study. We endorse this idea, although we demonstrate some of its limitations below. But how does one arrive at reasonable LGCM parameters needed to compute an index like ECR when no estimates from existing studies are available? In the next section, we offer some heuristics on how to go about solving this problem, given the limited information that is typically available at the time of study design.

Some heuristics for estimation of ECR need to include the separate contributions of different sources of error variance to effective error. The total error variance in a univariate LGCM is a sum of slope regression residual variance and indicator error variance. Slope regression residuals are errors of prediction in the usual sense of a regression equation, and their variance summarizes dispersion of occasion-specific latent factor scores around the best-fit line estimated by the LGCM (that is, they are occasion-specific shocks or disturbances in the latent variable space). Indicator error captures stochastic measurement error variance in the observed variable at each occasion. The distinction between these sources of error variance should be borne in mind when attempting to set parameter values in *a priori* power analysis. In practice, one usually has a static reliability estimate for a given measure that can be used to estimate measurement error variance, even though assumptions about measurement equivalence across time may not strictly hold in a given data set (Meredith and Horn, 2001). However, one also needs an estimate of regression residual variance. Our current approach to this problem invokes the assumption of homogeneous residual error variances as a heuristic that can be relaxed in more advanced applications. The following simple illustration of the underlying more complex logic exemplifies this.

Assume that information is available on the stability of individual differences of the target variable in a comparable population over a particular epoch of time. Given this information, provisional estimates of parameter values for the LGCM power analysis can be based on what is known about relations between stability of individual differences and LGCM parameters (Rogosa et al., 1982; Rogosa and Willett, 1983). In particular, there is a close correspondence between stability over time and ICC. In the following we show how the estimates of indicator reliability (such as parallel-forms reliability) and estimates of stability over time can be leveraged to estimate error variance in the LGCM for power analysis purposes.

Consider classical test theory approaches to alternate-forms reliability (e.g., Jöreskog, 1971). Under the assumption of homogeneous measurement error and no change between measurements, the correlation of these two measurements estimates their reliability (and corresponds to the intra-class correlation, ICC):

(12) $$\begin{array}{r} \rho_{reliability}\;=\;\frac{\sigma_{I}^{2}}{\sigma_{I}^{2}\;+\;\sigma_{\epsilon }^{2}} \end{array}$$

This allows us to relate the alternate-forms reliability to the individual differences in intercept and the residual error variance, which can be considered invariant over time for our purposes. If we are willing to assume a linear change process with true individual differences in change, then we can leverage a stability coefficient (a correlation of a variable with itself over time in a given sample (e.g., Bloom, 1964), sometimes labeled a test-retest correlation), to achieve estimates of intercept variance and regression residual variance. This approach is based on simplifying the following equation for the stability coefficient:

(13) $$\begin{array}{r} \rho_{stability}\;=\;\frac{\sigma_{I}^{2}+T\sigma_{IS}}{\sqrt{\sigma_{I}^{2}+\sigma_{\epsilon }^{2}}\sqrt{\sigma_{I}^{2}+\sigma_{\epsilon }^{2}+T^{2}\sigma_{S}^{2}+2T\sigma_{IS}}} \end{array}$$

Equations (12) and (13) allow us to translate stability and reliability estimates into growth curve parameter estimates and thus aid in study design planning by reducing degrees of freedom in the choice of parameter values. In the following, we present illustrative cases to show how these transformations can be applied, and more broadly, how design decision can be informed by using effective error and ECR.

## Illustrated applications

### Case 1: deriving LGCM weights

Here we will illustrate how the relationships identified in Equations (12) and (13) using minimal pre-existing information about the phenomena under investigation can generate useful parameter values for the evaluation of statistical power. Suppose a researcher is interested in longitudinally measuring episodic memory performance in a group of older adults. If a similar study has already been conducted via LGCM, we would generally be willing to use its estimates to generate a power analysis. For this purpose, we would assume generalizability in measures, procedures, occasions, and populations. But what if a comparable study with LGCM results is lacking, that is, there is neither a report of existing effect sizes nor of parameter values that could serve as a starting point for our analysis of change sensitivity? In a literature review, the researcher does find (a) a cross-sectional study reporting an alternate-forms reliability of 0.9 for the memory task, and (b) longitudinal data on a similar task reporting a 3-year stability coefficient of 0.85. These values reduce uncertainty about possible LGCM parameter values and can be leveraged to start the power analysis process.

In our example, we assume a scaling in T-score units, such that the total observed variance is 100 (given that *SD* = 10). If there were no individual differences in change (i.e., $\sigma_{S}^{2}=0$), there would be perfect stability of individual differences and hence zero regression residual error. In this limiting case, which we assume to hold with a parallel-forms reliability estimate, we find that $\sigma_{I}^{2}+\sigma_{E}^{2}=100$. Taking this information together with the other given, ρ_*reliability*_ = 0.9, and solving Equation (12), we see that $\sigma_{I}^{2}=90$ and $\sigma_{E}^{2}=10$. Of course, our focus is on variance in change, and if we assume true individual differences in change, then the 3-year test-retest stability estimate must be lower than the parallel-forms estimate because these systematic differences in rates of change contribute to the observed score variances.

We can apply Equation (13) to generate a crude estimate of $\sigma_{S}^{2}$. A practical constraint is that it is usually impossible to specify a point value for the intercept-slope covariance term σ_*IS*_ in advance. However, one solution to the problem is to assume that σ_*IS*_ = 0, at least as a starting point. With this assumption we can solve Equation (13) for $\sigma_{S}^{2}$ by inserting *T* = 3 and using our previous estimates of $\sigma_{I}^{2}$ and $\sigma_{E}^{2}$, yielding an estimate of $\sigma_{S}^{2}\;=\;1.35$. These parameter values can then be used to conduct statistical power analysis for a longitudinal design based on the specific parameters.

Brandmaier et al. (2015) developed a power analysis utility for LGCM slope variances called LIFESPAN. Using LIFESPAN (or the R code in the Supplementary Material) we can fill in the derived values for a design that is supposed to measure participants, for example, 3 times over 5 years, and we obtain estimated $\sigma_{eff}^{2}=0.76$, ECR = 0.64, and GRR = 0.63. The effective error gauges the precision of the design in units of unstandardized effect size without relying on a specific unstandardized effect size. The standardized effect size metrics indicate that a substantial amount of change-related variance in the variable of interest is associated with systematic individual differences in rates of change.

We might want to be more conservative when estimating true slope variance based on point estimates of residual variability and indicator reliabilities, given that these may be based on minimal information from the literature. For example, we might be targeting a slightly different population for which we believe change to be more difficult to detect (e.g., a sample that is slightly younger, slightly better educated, or that includes a lower proportion of participants with dementia) than the population generating available information on indicator reliability and stability. Table 1 presents a range of slope variance estimates for different test-retest stabilities crossed with different alternative-forms reliabilities. The smaller the test-retest stability, the smaller the proportion of stable individual differences and the larger the estimate of variance in change is.

**Table 1:** Slope variance as a function of reliability and stability.

**Reliability**	**Stability**				
	0.750	0.800	0.850	0.875	0.890
0.890	4.54	2.64	1.07	0.38	0.00
0.900	4.89	2.95	1.35	0.64	0.25
0.925	5.79	3.74	2.05	1.31	0.89

*Slope variance values are derived under the assumption of no intercept-slope covariance and no slope regression residuals*.

Table 1 highlights that there can be no true variance in slopes when both estimates of reliability and stability are identical. As noted by Rogosa et al. (1982), this is the degenerate case in which there are no individual differences in change, and in which the reliability of the difference score must be zero. In our terms, there is no opportunity for change sensitivity because there are no individual differences in change that the design must be able to detect.

In practice, engaging in power analysis means that we can entertain a range of possible slope variance effect sizes. Use of a power analysis program like LIFESPAN facilitates generation of a range of possible power values by systematically varying values of error variance components, but the same thing can also be done with values of ECR and unstandardized effect size (see Brandmaier et al., 2015).

### Case 2: evaluating and comparing study designs

Evaluating and comparing existing study designs is often a useful first step when planning a longitudinal study. When *post-hoc* power estimates are available from a set of pertinent studies (e.g., Rast and Hofer, 2014), one can call on these existing designs as a starting point for a future design. As argued earlier, statistical power can be decomposed into several components influencing change sensitivity. In the following example, we show how different indicators of change sensitivity may inform us in complementary and useful ways when comparing longitudinal studies with respect to their change sensitivity.

Assume we have singled out two existing studies from the literature targeting the same or a similar outcome of interest. In each, participants were measured every 3 months for 1 year and it happens that the estimated intercept variances were similar, suggesting an approximate $\sigma_{I}^{2}\;=\;90$. The studies fundamentally differ in the precision of measurement instruments used (5.9 in Study A and 10 in Study B), in the magnitude of estimated change variance (3.0 in Study A and 5.0 in Study B), and in sample size (200 participants in Study A and 300 in Study B). Now, a *post-hoc* power analysis reveals that Study A has a power of roughly 70% to reject the null hypothesis, $\sigma_{S}^{2}\;=\;0$, whereas Study B has a corresponding power of 85%. If presented with these power values only, we might be inclined to start our own study design by building upon the original design of the more powerful study, namely Study B. However, the difference in sample sizes between the studies must also be considered in evaluating the space of possible designs that are made up of alternative sample sizes, different temporal spacing of observations, and so on. As we noted earlier (see Figure 1), statistical power is determined by standardized effect size and sample size. Since we may not yet have decided on a sample size for our new study, it seems reasonable to compare the reliabilities of the studies given that reliabilities are independent of sample size. Using the LIFESPAN program, we find that for both studies, ECR = 0.246. Thus, one possible inference, based only on reliability, is that the difference in observed statistical power is solely due to the larger sample size in Study B. Upon closer inspection of the differences between the studies, we are reminded that the dependent variables also had different indicator reliabilities. Willett (1989) already noted that reliability *confounds* both individual differences in change and measurement precision, which may present hidden problems for the unwary investigator. If we wanted to plan our future study design independently of the absolute magnitude of individual differences, we can turn to effective error as a metric that is in units of (estimated) true effect size but independent of its magnitude. Doing so, we obtain $\sigma_{eff}^{2}$ = 9.20 for Study A and $\sigma_{eff}^{2}$ = 15.33 for Study B. By comparing effective error (which only works when variables are scaled in the same metric), we consider the measurement model and factor out sample size and true effect size. Then we find that Study A is the more sensitive measurement model (with more favorable precision and temporal spacing of measurements) to detect change variance despite its lower estimated *post-hoc* power even though Study B is the overall more powerful study when respective unstandardized effect size (magnitude of population slope variance) and sample sizes are also taken into account. This result may seem trivial as the difference in effective errors in this illustration—for the sake of simplicity—is solely due to apparent differences in indicator reliability. In actual applications, study differences in ECR may often reflect differences arising from constellations of total study duration, measurement density, and indicator reliability. Although we advocate using a standardized effect size measure like ECR in design planning, this example shows that no single measure of change sensitivity is inherently superior to the others; instead, they convey complementary summaries of study design properties.

### Case 3: finding optimal measurement density

Suppose a group of researchers is about to start a longitudinal study. The initial design calls for five occasions of measurement, each 1 year apart, thus spanning a total time of 4 years. Based on a literature review, the group assumes $\sigma_{I}^{2}\;=\;10$, $\sigma_{S}^{2}\;=\;1$, and $\sigma_{\epsilon }^{2}=50$. Using the LIFESPAN program, the researchers compute an effective error $\sigma_{eff}^{2}$ = 2.50 and a relatively low reliability of ECR = 0.29. When discussing the design, the question comes up whether a different spacing of measurements in time would be better to detect individual differences in change. The initial design will be denoted as a set of time points at which measurement takes place relative to study onset, {0, 1, 2, 3, 4}. The following alternative spacings of measurement occasions are discussed: for example, for organizational reasons, either the first three or last three measurement occasions could be moved closer together with time differences between measurements of 6 months each: {0, 0.5, 1, 3, 4}, {0, 1, 3, 3.5, 4}. Table 2 lists ECR, GRR, and Monte-Carlo-simulated power values for all three designs. Note that ECR values are scaled proportionally to statistical power whereas the two alternative designs have equal GRR values but different statistical power. The difference arises because the ICC_2_ = 10/(10 + 50/5) = 0.5, which is different from the asymptotic case of ICC_2_ = 1, which would render both effect size indices identical. ECR, not GRR, adequately quantifies differences in effective error due to the different spacing of measurement occasions. Given that sample size and unstandardized magnitude of individual differences in change are constant across all alternative designs considered, deciding upon the best (that is, the most change-sensitive) design can actually be based on effective error (coherent with ECR) alone and need not necessarily rely on simulated power values.

**Table 2:** Measures of study design quality for different distributions of five measurement occasions over 4 years.

**Design**	**Dispersion**	**GRR**	**ECR**	**Statistical power (*N* = 100)**
{0, 1, 2, 3, 4}	2.50	0.17	0.29	0.51
{0, 0.5, 1, 3, 4}	2.95	0.19	0.28	0.51
{0, 1, 3, 3.5, 4}	2.95	0.19	0.33	0.63

*Statistical power refers to the 1-df variance test and is based on a Monte Carlo estimate with 2,500 repetitions. Dispersion is given as the variance of the measurement time points. Note that the second and the third design have equal GRR values despite different ECR values and statistical power*.

## Discussion

This paper has demonstrated how measures of sensitivity to detect individual differences in linear change including statistical power can be leveraged to help researchers communicate and make decisions about longitudinal designs. Building on earlier results (von Oertzen, 2010; von Oertzen and Brandmaier, 2013), we have described how the central concept of effective error relates to concepts of reliability and standardized effect size for the LGCM slope variance parameter, captured either as GRR or ECR. The direct connection among effective error, standardized effect size, and statistical power of the 1-*df* LR test in a maximum likelihood framework provides a comprehensive perspective on sensitivity to detect individual differences in change that can be used as a benchmark for evaluating alternative longitudinal designs in terms of change sensitivity.

The developments presented here allow for a much broader and more comprehensive approach to analyzing, diagnosing, and planning longitudinal studies than is common practice in the field. In our view, both a lack of a formal system for comparison and contrast of influences of design decisions on statistical power, and the absence of a practically implementable approach to testing a wide range of alternative design configurations have led researchers to neglect *a priori* power analysis of change sensitivity at the planning stages of longitudinal investigations.

The approach illustrated here provides a means by which researchers can systematically and comprehensively consider trade-offs of design features that generate equivalent power. They can also examine how change sensitivity can be improved by attending to specific design features, such as adding occasions of measurement or prolonging the total study duration. Furthermore, this approach has been implemented in a power analysis utility program (LIFESPAN; Brandmaier et al., 2015) that facilitates iterative consideration of how power changes as a function of manipulating different design features. In the Supplementary Materials, we also provide R code to compute the discussed indices of precision and reliability. This paper provides a formal treatment of the elements that are essential to make this approach both statistically coherent and practically feasible. Of course, the LIFESPAN approach is a first pass at a utility aiding in the formalization of the optimal-design problem, and we fully expect that future efforts can greatly improve upon how the *a priori* design process can be supported by alternative optimization approaches.

Considerations of statistical power are inevitably a function of the specific hypotheses being tested. Researchers typically focus on more specific questions than detecting variance in change. Hence a generic assessment of change sensitivity is no substitute for an evaluation of a longitudinal design's power with respect to a more specific hypothesis critical to the viability of the respective study. Given that most longitudinal studies include multiple outcome variables, one would need to consider the possibility of a change sensitivity analysis within each relevant construct domain, an aspect of the problem that we do not consider further in this paper.

Building on previous work by Willett (1989) and Rast and Hofer (2014), we have shown that both ECR and GRR can be regarded as a metric-free index of slope variance effect size. We also showed that, when σ_*IS*_ = 0, GRR is a special case of ECR, and that only ECR is coherent with the effective error term for the specific and generalized LR test of zero slope variance that governs its power. Although GRR and ECR are closely related, we have shown here that ECR provides a better basis for capturing statistical power of the 1-*df* LR test (assuming no intercept-slope covariance) or the 2-*df* LR test (assuming an arbitrary intercept-slope covariance), because it does not ignore the contribution of intercept variance (scaled as ICC_2_) and intercept-slope covariance to the power of the LR test. On the other hand, GRR may be better suited for indexing an effect size coherent with the statistical power of the Wald test as it ignores intercept variance and intercept-slope covariance like the Wald test does. Along with others in the literature, we continue to argue (Hertzog et al., 2008) that the generalized 2-*df* test of zero slope variance is superior to the other tests, and generally preferable, because a non-zero intercept-slope covariance is also evidence of individual differences in change. Therefore there is a pressing need to extend the line of research presented in this paper to focus on how the more complex effective error for a 2-*df* LR test of slope variance—and an effect size index based upon it—may be analytically derived to achieve a better understanding about how design factors, individually and in interaction, determine statistical power to detect variance in change.

Previously, Rast and Hofer (2014) criticized simulations of LR test power attending to indices of error that were influenced by intercept variance (e.g., Hertzog et al., 2008) and advocated the merits of GRR for power analysis based on the fact that it ignored intercept variance. We have shown, to the contrary, that the power of the specific variance LR test is influenced by intercept variance (as scaled by ICC_2_) and that this influence on power is reflected in effective error and ECR, but not by GRR. The interconnection of intercept variance and slope variance can also be shown via the asymptotic non-independence of both components; the fact that the Fisher information matrix in an LGCM has non-zero entries for the covariance of the intercept variance parameter and slope variance parameter (even if intercept-slope-covariance is zero) violates the asymptotic independence assumption and has implications on bias in parameter estimates, standard errors, and statistical power (Kaplan and Wenger, 1993).

In general, we advocate that studies of LGCM begin to report estimates of ECR and GRR to provide a means to scale change sensitivity across different studies with different outcome variables^3^. One benefit of reliability indices such as ECR and GRR is that they allow for meta-analysis of effect sizes based on existing findings in the literature. Rast and Hofer (2014) used GRR to characterize existing studies in the field of normal cognitive aging. Our findings demonstrate another benefit: The availability of ECR as an effect size statistic will further improve the field's ability to conduct *a priori* power analyses that attend specifically to change sensitivity, often by leveraging information from other studies. Given that studies often do not report the full set of unconditional LGCM variance parameter estimates that can be used to generate estimates of ECR, we recommend that studies using linear LGCM begin reporting an estimate of ECR as a measure of slope effect size that can be useful for communication of results and can facilitate longitudinal design decisions by other researchers.

Although the ultimate target for evaluating change sensitivity is the statistical power to detect slope variance, statistical power is not the only index of change sensitivity to consider or report. Rather, our illustrative cases show that any of the three measures—effective error, ECR, or statistical power—can provide helpful information about aspects of change sensitivity. When examining alternative design possibilities within a single study, it seems reasonable to operate on the most basic component of change sensitivity proposed here, that is, the effective error of the slope variance test, as it already allows for the analysis of trade-offs of study design properties against each other. In particular, effective error enables researchers to analyze the effect of adding or removing occasions of measurement, prolonging or shortening the study's duration, or switching between differentially reliable measurement instruments (von Oertzen, 2010; von Oertzen et al., 2010; von Oertzen and Brandmaier, 2013). Reliability, as indexed by ECR, scales a candidate slope variance parameter by its effective error in a given design. As such, it provides a metric for standardized effect size that can be used to compare different study designs against each other directly and efficiently. Furthermore, one can consider absolute precision of the design in terms of effective error as well (see Case 2). Effective error is expressed in absolute units of unstandardized effect size but is independent of both sample size and true effect size.

In the study design phase, guesses about effect sizes may draw upon reports of published studies. If no plausible estimates of absolute magnitude of slope variance can be made when planning a longitudinal study, effective error is still applicable as a metric for comparisons of change sensitivity when deciding between different proposed study designs for the same outcome (i.e., when change is measured in the same units across designs). If longitudinal outcomes of different studies can legitimately be scaled to a common metric (e.g., T-scores), then effective error provides a metric for comparisons of relative precision even across different scales.

Another commonly reported measure of reliability in latent variable models is ICC_2_. ICC_2_ is closely connected to ECR but targets the reliability of intercept variance. It is usually defined as a reliability measure in which variance due to differences between persons in a repeated-measures design is expressed as a proportion of the total variance (Raudenbush and Bryk, 2002). ICC_2_ can be identically obtained by following the logic proposed here to derive reliability (ECR) in an intercept-only LGCM, in which no change of the construct is assumed. Then, we obtain ICC_2_ as the reliability of the intercept variance (representing stable between-person differences).

### Limitations

The limitations of using any of the indices developed in this paper inherit the well-known limitations of statistical power analysis in general. If all parameters of the LGCM can be specified *a priori* (i.e., they can be considered known at the time of planning), existing software such as Mplus (Muthen and Muthen, 2007), PinT (Snijders et al., 2007), OpenMx (Boker et al., 2011), simsem (Pornprasertmanit et al., 2016), or SIMR (Green and MacLeod, 2016) can be used to evaluate the power to reject candidate values of the parameter of interest—in our case, slope variance. For practical purposes, the need to specify values of parameters in advance can be an impediment to further efforts to use *a priori* power analysis to inform design decisions. At one extreme, this problem can be avoided by simply calculating the power to reject a particular magnitude of overall model fit using a relative goodness-of-fit index, as suggested by MacCallum et al. (2006). One can thereby make statements about the power of the entire model to detect consequential violations in overall model fit. However, this is not equivalent to evaluating power to reject a critical null hypothesis about a specific parameter. Our claim is that change sensitivity in a longitudinal study is often best captured by the ability to detect LGCM slope variance.

If *post-hoc* estimates of population values are used to determine indices of change sensitivity, the uncertainty about the empirical point estimates of the parameters induces uncertainty in the derived indices. That is, effective error, effective growth curve reliability, and statistical power reflect true change sensitivity only to the degree to which *post-hoc* parameter estimates involved in their computation (e.g., residual error, slope variance, or intercept variance) reflect the corresponding population values. The same is necessarily true for *a priori* guesses that can only be as accurate as the researcher's *a priori* knowledge or intuition in picking parameter values. Therefore, it is typically recommended that a designer choose conservative values during study design planning (von Oertzen and Brandmaier, 2013). In the light of uncertainty of best guesses, it is also advisable to calculate reliability of change or statistical power for a range of possible values and either to integrate over this range or to select the most conservative value among them. Alternatively, it may be useful to treat uncertainty in estimates formally (Kelley and Rausch, 2011; Lai and Kelley, 2011; Gribbin et al., 2013) and to derive confidence intervals for effect size estimates in accordance with best practices proposed by Kelley and Preacher (2012). Future work should focus on capturing the uncertainty around single-point estimates of ECR and GRR.

In a similar vein, effective error, reliability, and statistical power are only valid under the assumption that the growth process is adequately modeled, that is, there is no misspecification in the underlying LGCM. The perfect interchangeability of power-equivalent operations—for instance between adding more occasions within a given total study duration or increasing total study duration—is based on the assumption that linearity of change is strictly true, and not just a useful approximation for shorter stretches of time. Also note that our considerations are only valid under the assumption of homogeneous residual error variances over time. The present manuscript is a central building block for planned future work addressing more complex models, such as non-linear growth curve models or (dual) change score models, and other types of hypotheses (e.g., the generalized variance test). We recommend to use Monte-Carlo-based simulation approaches (Muthén and Muthén, 2002) for assessing more general designs currently not covered by our approach. However, simulation-based approaches will always be slower and their results will always be more difficult to generalize than an analytical solution that allows a fuller overall theoretical understanding of what is being studied. For example, solving the problem of finding alternative, power-equivalent designs to an initial study design (Brandmaier et al., 2015) may be time-consuming, at best, if not infeasible when purely relying on simulation-based approaches.

Finally, missing data, and specifically attrition in longitudinal data, decrease the change sensitivity of longitudinal designs. von Oertzen and Brandmaier (2013; Appendix B, Theorem 4) proved that under a missing-completely-at-random (MCAR) assumption, an effective error can be derived for each group that has a unique attrition pattern, and the resulting effective error is a weighted harmonic mean of the effective errors aggregated over attrition groups. Given that attrition from longitudinal studies is decidedly not MCAR, further work on this problem is needed. With the same approach, spacing between measurements varying both within and across people can be accounted for by conceiving of such a study design as a multiple-group design, in which each person is his or her own group, and in which the overall effective error is a function of the person-specific effective errors.

Future work should also address additional statistical issues. The power equivalence theory that is the basis for the present work assumes that all parameters other than the values subject to a hypothesis test are known and fixed. Power-equivalent operations are performed under this assumption; however, in practice, all LGCMs are typically freely estimated from data. The same assumption is made in other analytical approximations of statistical power, such as that of Satorra and Saris (1985). When researchers are exploring a design space for variations in change sensitivity, they need to be aware that the LR test in question may have lower power than is estimated by the power equivalence approach because it actually freely estimates all parameters. Thus, after a candidate design has been chosen using power-equivalence trade-offs, a final Monte Carlo simulation to generate a more accurate estimate of power with the candidate design should be conducted. The LIFESPAN program can generate the Monte Carlo simulation for a specified set of hypothetical model parameters generated by power-equivalence searches of the design-trade-off space (Brandmaier et al., 2015).

To conclude, we believe that the notions of precision (scaled as effective error), reliability, and statistical power help to promote the insight that increasing sample size is not the only, and not necessarily even the best way to optimize change sensitivity of a longitudinal design. Instead, all design decisions influence change sensitivity, and research designs can be optimized accordingly even before the size of the sample has been specified. Greater sample size increases statistical power of the LR test, to be sure, but one can consider other means to achieve this that may be more practically feasible in a given study context (e.g., by administering instruments with higher reliability). The present approach enables researchers to evaluate, optimize, and communicate longitudinal designs comprehensively by considering how design features interactively influence change sensitivity in LGCM.

## Author contributions

All authors contributed to this manuscript. AB and TvO derived the mathematical proofs in the Supplementary Material.

### Conflict of interest statement

The authors declare that the research was conducted in the absence of any commercial or financial relationships that could be construed as a potential conflict of interest.

## Footnotes

^1^We are not referring here to equivalent models, that is, cases in which models with different parameter specifications generate identical derived mean and covariance structures (Stelzl, 1986; Lee and Hershberger, 1990; MacCallum et al., 1993). Instead, we are referring to models that, even if qualitatively dissimilar in terms of model parameter specification, generate identical effective error for the effect of interest.

^2^Note that this likelihood ratio is equal to the likelihood ratio of the original model from which the power-equivalent minimal model was derived (see Figure 2 for illustration), with a zero-slope restriction on the original model (see von Oertzen, 2010).

^3^An estimate of ECR from a LGCM using ML estimation is obtained by substituting estimates of LGCM parameters in Equation (7). Currently, this can be accomplished by inputting LGCM parameter estimates into the LIFESPAN program, which returns values for effective error, ECR, and GRR.
