# Supplementary material for: Precision, Reliability, and Effect Size of Slope Variance in Latent Growth Curve Models: Implications for Statistical Power Analysis
Source: Front Psychol. 2018 Apr 17;9:294. doi: 10.3389/fpsyg.2018.00294 (PMC5932409; doi:10.3389/fpsyg.2018.00294)
Supplement: Supplementary file 1 [file DataSheet1.PDF]

Supplementary Material: Precision, Reliability, and Effect Size of Slope Variance in Latent Growth Curve Models: Implications for Statistical Power Analysis

Andreas M. Brandmaier*, Timo von Oertzen, Paolo Ghisletta, Ulman Lindenberger and Christopher Hertzog
*Correspondence:
Author Name: Andreas M. Brandmaier
brandmaier@mpib-berlin.mpg.de

1 CONTRIBUTORS TO EFFECTIVE ERROR IN LATENT GROWTH CURVE MODELS

Let us assume a univariate LGCM, as defined in the main body of the article (Equation 1). Let σ^2_ϵ be the residual error, σ^2_I the intercept variance, ρ the covariance between intercept and slope, and M the total number of measurement occasions at time points $t_1, \ldots, t_T$. We assume slope weights t_j to be equal to the observed time points; in other cases, replace t_j in all Equations with rescaled terms. Under the assumption of $\rho = 0$, von Oertzen and Brandmaier (2013) have shown that for arbitrary but known choices of σ^2_ϵ, σ^2_I, M, and $t_1, \ldots, t_M$, there is a power-equivalent minimal SEM in which the slope is directly observed with some complex residual error variance, referred to as *effective error*. This hypothetical model has the same power on a likelihood ratio test to detect a non-zero slope variance as the original design. In their Appendix A, von Oertzen and Brandmaier (2013) provide the following analytical solution for the effective error:

$$\sigma^2_{eff} = \frac{\sigma^2_\epsilon \left(\sigma^2_I M + \sigma^2_\epsilon\right)}{\left(\sigma^2_I M + \sigma^2_\epsilon\right) \sum_{j=1}^{M} t_j^2 - \sigma^2_I \left(\sum_{j=1}^{M} t_j\right)^2} \tag{S1}$$

In the following, we extend and generalize Appendix A of von Oertzen and Brandmaier (2013), to derive the more general solution for any known and fixed covariance ρ between intercept and slope.

THEOREM 1. *Let $\sigma^2_I, \sigma^2_\epsilon, M$, and all t_i be as above. The effective error, σ^2_{eff}, for hypothesis tests on the latent slope in univariate linear LGCMs under the assumption of a known and fixed covariance ρ between latent intercept and latent slope is:*

$$\sigma^2_{eff} = \frac{\sigma^2_\epsilon}{\sum_{j=1}^{M} t_j^2 - \eta \left(\sum_{j=1}^{M} t_j\right)^2} + \zeta \tag{S2}$$

with

$$\zeta = \frac{\rho^2 \left(\left(\sum_{j=1}^M t_j \right)^2 - M \left(\sum_{j=1}^M t_j^2 \right) \right) + 2\rho \left(\sum_{j=1}^M t_j \right) \sigma_\epsilon^2}{\left(\sum_{j=1}^M t_j^2 \right) \left(M\sigma_I^2 + \sigma_\epsilon^2 \right) - \left(\sum_{j=1}^M t_j \right)^2 \sigma_I^2}$$

and

$$\begin{aligned} \eta &= \frac{1}{\left(M + \sigma_\epsilon^2 / \sigma_I^2 \right)} \\ &= \frac{1}{\frac{M\sigma_I^2 + \sigma_\epsilon^2}{\sigma_I^2}} \\ &= \frac{\sigma_I^2}{M\sigma_I^2 + \sigma_\epsilon^2} \\ &= \frac{1}{M} \cdot \frac{\sigma_I^2}{\sigma_I^2 + \frac{\sigma_E^2}{M}} \\ &=: \frac{ICC_2}{M} \end{aligned}$$

PROOF. Using successive applications of power-equivalent transformations (see von Oertzen (2010); also see von Oertzen and Brandmaier (2013) for an analogous derivation), we obtain a minimal power-equivalent model with the same latent structure as the original model. The equivalent model can be represented in terms of a symmetric latent covariance matrix $\mathbf{C}$, a symmetric matrix with regression weights from latent variables to observed variables $\mathbf{\Lambda}$ and a residual error covariance matrix $\mathbf{E}$

$$\mathbf{\Lambda} = \begin{pmatrix} 1 & 0 \\ \lambda & 1 \end{pmatrix} \quad \mathbf{C} = \begin{pmatrix} \sigma_I^2 & \rho \\ \rho & \sigma_S^2 \end{pmatrix} \quad \mathbf{E} = \begin{pmatrix} e_1 & 0 \\ 0 & e_2 \end{pmatrix}$$

with the following composite terms:

$$\begin{aligned} \lambda &= \frac{\sum_{j=1}^M t_j}{\sum_{j=1}^M t_j^2} \\ e_1 &= \frac{\sigma_\epsilon^2}{M - \left(\sum_{j=1}^M t_j \right)^2 / \sum_{j=1}^M t_j^2} \\ e_2 &= \frac{\sigma_\epsilon^2}{\sum_{j=1}^M t_j^2} \end{aligned} \tag{S3}$$

The model-implied observed covariance matrix, $\mathbf{\Sigma} = \mathbf{\Lambda} \mathbf{C} \mathbf{\Lambda}^T + \mathbf{E}$, can be written as

$$\boldsymbol{\Sigma} = \begin{pmatrix} \sigma_I^2 + e_1 & \lambda\sigma_I^2 + \rho \\ \lambda\sigma_I^2 + \rho & \sigma_S^2 + \lambda^2\sigma_I^2 + 2\lambda\rho + e_2 \end{pmatrix} \tag{S4}$$

$$= \underbrace{\begin{pmatrix} \sigma_I^2 + e_1 & \lambda\sigma_I^2 + \rho \\ \lambda\sigma_I^2 + \rho & \lambda^2\sigma_I^2 + 2\lambda\rho + e_2 \end{pmatrix}}_{\Sigma_E} + \underbrace{\begin{pmatrix} 0 & 0 \\ 0 & \sigma_S^2 \end{pmatrix}}_{\Sigma_S} \tag{S5}$$

Equation S5 is a partition of $\boldsymbol{\Sigma}$ into a signal part $\boldsymbol{\Sigma}_S$, containing the variance contributions of the slope, and a noise part $\boldsymbol{\Sigma}_E$, which contains nuisance contributions only. A Cholesky decomposition of $\boldsymbol{\Sigma}_E$ allows to express $\boldsymbol{\Sigma}$ as

$$\boldsymbol{\Sigma} = \sqrt{\boldsymbol{\Sigma}_E}\sqrt{\boldsymbol{\Sigma}_E}^T + \boldsymbol{\Sigma}_S$$

with

$$\sqrt{\boldsymbol{\Sigma}_E} = \begin{pmatrix} \sqrt{\sigma_I^2 + e_1} & 0 \\ \frac{\lambda\sigma_I^2+\rho}{\sqrt{\sigma_I^2+e_1}} & \sqrt{\lambda^2\sigma_I^2 + 2\lambda\rho + e_2 - \frac{(\lambda\sigma_I^2+\rho)^2}{\sigma_I^2+e_1}} \end{pmatrix}$$

By applying the power-equivalent deletion rule (see von Oertzen (2010), rule 5 “deletion”), we obtain a power-equivalent model of a latent slope and a single effective error variance, given by the square of the lower right entry of $\sqrt{\boldsymbol{\Sigma}_E}$:

$$\sigma_{eff}^2 = \frac{e_2\sigma_I^2 + e_1\lambda^2\sigma_I^2 + e_1e_2 + 2\lambda\rho e_1 - \rho^2}{\sigma_I^2 + e_1} \tag{S6}$$

Inserting the definitions of λ, e_1 and e_2 from Equation S3 and assuming assuming $\sum t_j := \sum_{j=1}^M t_j$ and $\sum t_j^2 := \sum_{j=1}^M t_j^2$, we obtain

$$\begin{aligned} \sigma_{eff}^2 &= \frac{\rho^2\left(\left(\sum t_j\right)^2 - M\sum t_j^2\right) + \left(2\rho\sum t_j + M\sigma_I^2\right)\sigma_\epsilon^2 + \left(\sigma_\epsilon^2\right)^2}{\sum t_j^2\sigma_\epsilon^2 + \left(M\sum t_j^2 - \left(\sum t_j\right)^2\right)\sigma_I^2} \\ &= \underbrace{\frac{\rho^2\left(\left(\sum t_j\right)^2 - M\sum t_j^2\right) + 2\rho\sum t_j\sigma_\epsilon^2}{\sum t_j^2\sigma_\epsilon^2 + \left(M\sum t_j^2 - \left(\sum t_j\right)^2\right)\sigma_I^2}}_{\zeta} + \underbrace{\frac{M\sigma_I^2\sigma_\epsilon^2 + \left(\sigma_\epsilon^2\right)^2}{\sum t_j^2\sigma_\epsilon^2 + \left(M\sum t_j^2 - \left(\sum t_j\right)^2\right)\sigma_I^2}}_{\xi} \end{aligned}$$

We can rewrite the above equation as sum of a part containing all ρ-terms, which we denote ζ, and one containing no ρ-terms, which we refer to as ξ. The latter can be simplified to

$$\begin{aligned}\xi &= \frac{M\sigma_I^2\sigma_\epsilon^2 + \left(\sigma_\epsilon^2\right)^2}{\sum t_j^2\sigma_\epsilon^2 + \left(M\sum t_j^2 - \left(\sum t_j\right)^2\right)\sigma_I^2} \\ &= \frac{\sigma_\epsilon^2\left(M\sigma_I^2 + \sigma_\epsilon^2\right)}{\sum t_j^2\left(M\sigma_I^2 + \sigma_\epsilon^2\right) - \sigma_I^2\left(\sum t_j\right)^2} \\ &= \frac{\sigma_\epsilon^2}{\sum t_j^2 - \eta\left(\sum t_j\right)^2} \end{aligned} \tag{S7}$$

Note that under the assumption of no correlation between intercept and slope, $\rho = 0$, and thus $\zeta = 0$, we obtain a simplified expression for the effective error, which was previously derived by von Oertzen and Brandmaier (2013) and is identical to Equation S1:

COROLLARY 2. *Let $\sigma_I^2, \sigma_\epsilon^2, M, \eta$, and t_j be as above. Under the assumption of no covariance between intercept and slope, i.e., $\rho = 0$, the effective error in a univariate, linear LGCM is*

$$\sigma_{eff}^2 = \frac{\sigma_\epsilon^2}{\sum_{j=1}^M t_j^2 - \eta\left(\sum_{j=1}^M t_j\right)^2}$$

Willett (1989) defined an observed growth-rate measure, $\hat{\theta_p}$, which he obtained by regressing, via OLS, observed status on time separately for each individual p (p. 590). He further deduced the within-individual sampling variation of the individual observed growth rates (p.591), which we call the effective error, to be:

$$\sigma_{Willett}^2 = \frac{\sigma_\epsilon^2}{\sum_{j=1}^M \left(t_j - \bar{t}\right)^2} \tag{S8}$$

for $\bar{t}$ being $\sum_{j=1}^M \frac{t_j}{M}$.

With the following corollary, we show that Willett's effective error is a special case of the effective error (von Oertzen and Brandmaier, 2013) that makes an additional approximation by assuming that the error variance can be neglected compared to the intercept variance, which can be evaluated in terms of a reliability measure of the intercept, ICC_2.

COROLLARY 3. *Let $\sigma_I^2, \sigma_\epsilon^2, M, \eta, \rho$ and t_i be as above. The error term of GRR (Willett, 1989) , $\sigma_{Willett}^2$, is a limit case of the effective error, σ_{eff}^2 if $\rho = 0$ and $ICC_2 = 0$ and $\sum_{j=1}^M t_j = 0$*

PROOF. We can equivalently rewrite Equation S1 by inserting η

$$\sigma_{eff}^2 = \frac{\sigma_\epsilon^2}{\sum_{j=1}^n t_j^2 - \frac{ICC_2}{M}\left(\sum_{j=1}^M t_j\right)^2} \tag{S9}$$

Furthermore, we can re-express $\sum_{j=1}^M \left(t_j - \bar{t}\right)^2$ from Equation S8 as $\sum_{j=1}^M \left(t_j - \bar{t}\right)^2 = M \cdot Var(t)$ and expand $Var(t)$ according to the rule $Var(X) = E(X^2) - E(X)^2$ to obtain

$$\begin{aligned}
\sigma^2_{Willett} &= \frac{\sigma^2_\epsilon}{\sum_{j=1}^{M}(t_j - \bar{t})^2} \\
&= \frac{\sigma^2_\epsilon}{M\left[\frac{1}{M}\sum_{j=1}^{M} t_j^2 - \frac{1}{M^2}\left(\sum_{j=1}^{M} t_j\right)^2\right]} \\
&= \frac{\sigma^2_\epsilon}{\sum_{j=1}^{M} t_j^2 - \frac{1}{M}\left(\sum_{j=1}^{M} t_j\right)^2} \qquad \text{(S10)}
\end{aligned}$$

Comparing Equations S9 and S10, the similarity of both effective error definitions becomes apparent. Note that σ^2_{eff} and $\sigma^2_{Willett}$ are identical iff $ICC_2 = 1$ or $\sum_{j=1}^{M} t_j = 0$.

To reiterate, $\sigma^2_{Willett}$ is a limit case of σ^2_{eff} under the assumption of no covariance between intercept and slope and if either $ICC_2 = 1$ or the time points of measurement are centered.

2 THE RELATION OF RELIABILITY AND STATISTICAL POWER

Here we demonstrate the relation of ECR to statistical power in a 1-df test about the slope variance in a linear LGCM.

THEOREM 4. *The non-centrality parameter λ is a function of sample size, N, and ECR:*

$$\lambda = N\left[\frac{1}{1-ECR} - \ln\left(\frac{1}{1-ECR}\right) - 1\right]$$

PROOF. Note that

$$\begin{aligned}
\frac{\sigma^2_S}{\sigma^2_{eff}} + 1 &= \frac{\sigma^2_S + \sigma^2_{eff}}{\sigma^2_{eff}} \\
&= \left(\frac{\sigma^2_{eff}}{\sigma^2_S + \sigma^2_{eff}}\right)^{-1} \\
&= \left(1 - \frac{\sigma^2_S}{\sigma^2_S + \sigma^2_{eff}}\right)^{-1} \\
&= (1 - ECR)^{-1}
\end{aligned}$$

The log-likelihood ratio, λ, between Σ_{res} and Σ_{min} is given by

$$\begin{aligned}
\lambda &= N\left[\ln\left(|\Sigma_{res}|\right) - \ln\left(|\Sigma_{min}|\right) + Tr\left(\Sigma_{res}^{-1}\Sigma_{min}\right) - 1\right] \\
&= N\left[\ln\left(\frac{\sigma_{eff}^2}{\sigma_S^2+\sigma_{eff}^2}\right) + \frac{\sigma_S^2+\sigma_{eff}^2}{\sigma_{eff}^2} - 1\right] \\
&= N\left[\ln\left(\frac{\sigma_{eff}^2}{\sigma_S^2+\sigma_{eff}^2}\right) + \frac{\sigma_S^2}{\sigma_{eff}^2}\right] \qquad \text{(S11)} \\
&= N\left[\frac{\sigma_S^2}{\sigma_{eff}^2} - \ln\left(\frac{\sigma_S^2}{\sigma_{eff}^2}+1\right)\right] \qquad \text{(S12)} \\
&= N\left[\frac{1}{1-ECR} - \ln\left(\frac{1}{1-ECR}\right) - 1\right] \qquad \text{(S13)}
\end{aligned}$$

3 GENERALIZED ECR

To obtain the ECR for LGCMs with non-zero intercept-slope-covariance, we compute the expected $M \times M$ covariance matrix Σ and the expected $M \times M$ covariance matrix Σ_0 of a model for which both intercept-slope-covariance and slope variance are set to 0. We compute the non-centrality parameter as follows:

$$\lambda = N\left[\log\left(\det\Sigma_0\right) + tr\left(\Sigma_0^{-1}\Sigma\right) - \log\left(\det\Sigma\right) - M\right]$$

Using a numeric one-dimensional optimization algorithm, e.g., golden section search and successive parabolic interpolation, bracketing the interval between 0 and 1, we invert the Equation from Theorem 4, to obtain ECR given N and λ by finding the root of:

$$\text{argmin}_{\text{ECR}}\left(\lambda - N\left[\frac{1}{1-ECR} - \ln\left(\frac{1}{1-ECR}\right) - 1\right]\right)^2$$

REFERENCES

von Oertzen, T. (2010). Power equivalence in structural equation modelling. *British Journal of Mathematical and Statistical Psychology* 63, 257–272. doi:10.1348/000711009X441021

von Oertzen, T. and Brandmaier, A. M. (2013). Optimal study design with identical power: An application of power equivalence to latent growth curve models. *Psychology and Aging* 28, 414–428. doi:10.1037/a0031844

Willett, J. B. (1989). Some results on reliability for the longitudinal measurement of change: Implications for the design of studies of individual growth. *Educational and Psychological Measurement* 49, 587–602. doi:10.1177/001316448904900309
