# Supplementary material for: Precision, Reliability, and Effect Size of Slope Variance in Latent Growth Curve Models: Implications for Statistical Power Analysis
Source: Front Psychol. 2018 Apr 17;9:294. doi: 10.3389/fpsyg.2018.00294 (PMC5932409; doi:10.3389/fpsyg.2018.00294)
Supplement: Supplementary file 2 [file DataSheet2.PDF]

```r
#
# Supplementary R code for
#
# Brandmaier, A.M., von Oertzen, T., Ghisletta, P., Lindenberger, U., & Hertzog, U.
# Precision, Reliability, and Effect Size of Slope Variance in Latent Growth Curve Models:
# Implications for Statistical Power Analysis
#
# [Submitted to Frontiers]
#
# Last edited: 2017, 12 DEC
#
# Contact: Andreas Brandmaier <brandmaier@mpib-berlin.mpg.de>
#
# The following R codes allow to compute various indices of reliability and effective error
# as described in the manuscript. For power simulations, please refer to R packages such
# as simsem or others, or directly use our graphical frontend LIFESPAN
# (http://www.brandmaier.de/lifespan)
#
#
# Examples
#
# 1) Specify a linear growth curve model with three measurements over five years
# (see Case #1 in the manuscript)

# model <- lgcm(timepoints = c(0,2.5,5), intercept.variance = 90, residual.variance = 10,
#     slope.variance = 1.35)

# 2) Compute effective error

# effective.error(model)
#
# [1] 0.759322

# 3) Compute ICC2

# icc2(model)
#
# [1]  0.9642857

# 4) Compute GRR

# grr(model)
#
# [1] 0.627907

# 5) Compute ECR (for slope variance)

# ecr(model)
#
# [1]  0.6400161


# ------------ functions -----------------

require("OpenMx")

lgcm <- function(timepoints=0, intercept.variance=0,
                 slope.variance=0,
                 residual.variance=0,
                 intercept.slope.covariance = 0)
{
  lgcm <- list()
  lgcm$timepoints <- sort(timepoints)
  lgcm$intercept.variance <- intercept.variance
  lgcm$slope.variance <- slope.variance
  lgcm$residual.variance <- residual.variance
  lgcm$total.study.time <- lgcm$timepoints[length(lgcm$timepoints)]
```

```
  lgcm$intercept.slope.covariance <- intercept.slope.covariance

  lgcm$sumti <- sum(lgcm$timepoints)
  lgcm$sumtisq <- sum(lgcm$timepoints^2)
  lgcm$num.timepoints <- length(timepoints)

  return(lgcm)
}

ecr <- function(lgcm)
{
  return (lgcm$slope.variance / (lgcm$slope.variance+effective.error(lgcm)))
}

grr <- function(lgcm)
{
  err <-  lgcm$residual.variance/( lgcm$sumtisq -
(1/lgcm$num.timepoints)*lgcm$sumti*lgcm$sumti)
  return (lgcm$slope.variance / (lgcm$slope.variance+err))
}

icc2 <- function(model)
{
model$intercept.variance/(model$intercept.variance+model$residual.variance/model$num.timepo
ints)
}


effective.error <- function(lgcm)
{

  eta <- 1 / ((lgcm$num.timepoints+ lgcm$residual.variance / lgcm$intercept.variance))

  rho <-  lgcm$intercept.slope.covariance

  zeta <- rho*rho*(lgcm$sumti*lgcm$sumti-lgcm$num.timepoints*lgcm$sumtisq)+
    2*rho*lgcm$sumti*lgcm$residual.variance
  zeta <- zeta /
    (lgcm$sumtisq*(lgcm$num.timepoints*lgcm$intercept.variance+lgcm$residual.variance)-
       lgcm$sumti*lgcm$sumti*lgcm$intercept.variance)
lgcm$residual.variance/( lgcm$sumtisq - eta*lgcm$sumti*lgcm$sumti)+ zeta
}
```
